# Improved Digit Span in Children after a 6-Week Intervention of Playing a Musical Instrument: An Exploratory Randomized Controlled Trial

**DOI:** 10.3389/fpsyg.2017.02303

**Published:** 2018-01-08

**Authors:** Xia Guo, Chie Ohsawa, Akiko Suzuki, Kaoru Sekiyama

**Affiliations:** ^1^Graduate School of Social and Cultural Sciences, Kumamoto University, Kumamoto, Japan; ^2^Faculty of Music, Kyoto City University of Arts, Kyoto, Japan; ^3^Kokoro Research Center, Kyoto University, Kyoto, Japan; ^4^Division of Cognitive Psychology, Faculty of Letters, Kumamoto University, Kumamoto, Japan; ^5^Graduate School of Advanced Integrated Studies in Human Survivability, Kyoto University, Kyoto, Japan

**Keywords:** working memory, executive function, cognition, instrumental music training, keyboard harmonica, children, short-term, randomized controlled trial

## Abstract

Previous studies have reported that music training not only improves children's musical skills, but also enhances their cognitive functions. However, there is a disagreement about what domain(s) might be affected. Moreover, effects of short-term (<several months) instrumental training have not been examined, although more basic studies have suggested neuroplasticity within several weeks. Consequently, the present exploratory pilot study investigated the effect of a six-week instrumental practice program (i.e., playing the keyboard harmonica) on children's cognitive functions using a randomized controlled trial. Forty children (aged 6–8 years) were randomly assigned to either the experimental group (*n* = 20), which received a 6-week (12-session) keyboard harmonica curriculum, or an untrained control group (*n* = 20). Different from traditional instrumental training, the curriculum did not use musical scores to emphasize creating association between sound (auditory modality) and finger movement (somato-motor system). Cognitive measurements included verbal ability, processing speed, working memory, and inhibitory control, which were administered before and after the curriculum in both groups. After the 6-week training, only the experimental group showed a significant improvement in the Digit Span test (especially in the Digit Span Backward) that measures working memory. However, no significant influences were found on the other cognitive tests. The result suggests that several weeks of instrumental music training may be beneficial to improving children's working memory. In addition, we used an inexpensive and portable keyboard harmonica; therefore, our instructional method is easy to apply in classrooms or other circumstances. If the method is applied to music lessons in schools or in the community, it may help improve children's working memory.

## Introduction

The composition of music is very complex; it contains tone, rhythm, harmony, melody, and other factors. Playing a musical instrument requires reading musical scores and translating them into motor commands, as well as performing coordinated movements and creating memory of musical phrases. Therefore, wide ranges of brain regions are active when individuals perform music activities (Levitin, 2006). In this context, many parents let their children learn musical instruments to try and improve their cognitive abilities through musical training (Schellenberg, 2004). We examined how instrumental music training influences more general cognitive abilities in a relatively brief period.

Several studies have shown an association between music training and improvement in cognitive skills. Cross-sectional studies have shown that musicians are superior to non-musicians on verbal (Chan et al., 1998; Forgeard et al., 2008; Chobert et al., 2011), spatial (Helmbold et al., 2005; Stoesz et al., 2007), mathematical (Cheek and Smith, 1999; Gouzouasis et al., 2007), and general cognitive abilities (Schellenberg, 2011). For example, Schellenberg (2011) revealed that musically trained children had a higher general intelligence (Wechsler Abbreviated Scale of Intelligence; Wechsler, 1999) than did untrained children, and this difference remained when demographic variables (parents' education, parents' first language, family income, and non-musical out-of-school activities) were held constant.

Consistent with these results, many brain imaging studies have revealed differences between musicians and non-musicians. In a neuroanatomical study, Schlaug et al. (1995a) found that, compared to non-musicians, musicians who began music training before the age of 7 years had a larger corpus callosum, which is a communication path connecting the left and right hemispheres of the cerebrum. Brain structure studies have reported structural differences between musicians and non-musicians in areas such as planum temporale (Schlaug et al., 1995b; Zatorre et al., 1998; Keenan et al., 2001), Heschl's gyrus (primary auditory cortex) (Gaser and Schlaug, 2003; Schneider et al., 2005), Broca's area (Sluming et al., 2002), and the inferior frontal gyrus (Gaser and Schlaug, 2003). A neuroimaging study using diffusion tensor imaging reported that number of hours of piano practice during childhood was positively correlated with increased fractional anisotropy (Bengtsson et al., 2005). Perhaps these differences can explain the increases in more general cognitive functions among musicians.

However, because most of these studies were cross-sectional, a causal relationship between music training and cognitive functions was not demonstrated. Consequently, more compelling evidence of musical training comes from randomized controlled trials (RCTs) (Costa-Giomi, 1999; Schellenberg, 2004; Fujioka et al., 2006; Moreno et al., 2011) and longitudinal correlational studies (Roden et al., 2012, 2014). One RCT (Schellenberg, 2004) demonstrated that 6-year-old children who received 36-weeks of keyboard or vocal lessons had a significantly greater increase in general intelligence (WISC-III; Wechsler, 1991) than children receiving drama lesson or no lessons at all. Another RCT over 3 years with 9-year-old children by Costa-Giomi (1999) found that the “piano group” improved significantly more than a matched control group on the Developing Cognitive Ability Test after 2 years; however, there was no difference between the two groups after 2 years.

Several experimental studies have addressed the causal relationship between instrumental training and children's cognitive functions, and most of them are based on the effects of long-term instrumental music training (Table 1). On the other hand, more basic studies have demonstrated that even short-term training or novel experience can induce neuroplasticity changes (Sekiyama et al., 2000; Scholz et al., 2009; Landi et al., 2011; Debowska et al., 2016), however, none of them focused on instrumental training. Therefore, we conducted an exploratory RCT to investigate the influence of short-term instrumental training on verbal ability, processing speed, working memory (WM), and inhibitory control in children.

**Table 1:** Review of previous randomized controlled trial/longitudinal studies evaluating effects of instrumental music training on child cognitive function.

**Study**	**Costa-Giomi, 1999**	**Schellenberg, 2004**	**Roden et al., 2012**	**Roden et al., 2014**
Experimental design	Randomized controlled trial	Randomized controlled trial	Longitudinal study	Longitudinal study
Age (years)	9	6	7.54	7.73
Final sample size	67	132	73	50
Group type	Private piano lesson vs. no lesson	Music (keyboard or voice) vs. control (drama or no lesson)	Instrumental (guitar, cello, etc.) vs. natural science vs. no lesson	Instrumental (guitar, cello, etc.) vs. natural science
Training length	3 years	36 weeks	18 months	18 months
Measures	Developing Cognitive Abilities Test Beggs and Mouw, 1980; Musical Aptitude Profile Gordon, 1988; Bruininks-Oseretsky Test of Motor Proficiency Bruininks, 1978; Canadian Achievement Test2 Canadian Test Centre, 1992; Coopersmith Self-Esteem Inventories Coopersmith, 1981	Wechsler Intelligence Scale for Children (WISC-III, Wechsler, 1991); Kaufman Test of Educational Achievement Kaufman and Kaufman, 1985; Behavioral Assessment System for Children Reynolds and Kamphaus, 1992	Verbal learning, Delayed recall, Verbal recognition Helmstaedter et al., 2001; Corsi Block, Matrix Span Hasselhorn et al., 2012	Corsi Block, One-syllable Word Span, Matrix Span, Couning Span, Complex Span, Color Span Backwards Hasselhorn et al., 2012
Main findings	Piano group showed significant improvement in Developing Cognitive Ability Test after two years; however, there were no differences between the two groups after three years.	Music group showed greater increase in full-scale IQ than did the control group.	Instrumental group showed greater improvements on verbal memory (Verbal learning, Delayed recall, and Verbal recognition) than did the natural science group and the control group.	Instrumental group showed greater improvements on phonological loop and central executive of working memory than did the control group.

Although a series of behavioral studies have reported associations between musical training and cognitive functioning, it is still unclear what cognitive domains are improved. When examining the Vocabulary subtest of the Wechsler Intelligence Scale for Children–Third Edition (WISC-III) (Wechsler, 1991), the scores of musically trained children were significantly higher than untrained controls; however, musically trained children did not outperform the control counterparts on phonemic awareness or spatial skills (Forgeard et al., 2008). Degé and Schwarzer (2011) reported that children who received 20-weeks of music program or the phonological skills program had a significantly greater increase in phonological awareness than children receiving sports training. As mentioned above, the results are mixed and what domain(s) might be affected are still unknown.

Both music and verbal abilities are high-level cognitive functions, which also have a lot of similarities (see Piro and Ortiz, 2009). Therefore, exploration of the relationship between music and verbal abilities has received attention from many researchers. A meta-analysis based on correlational studies showed a significant positive correlation between music education and reading tests, whereas the meta-analysis of the 6 experimental studies showed no reliable effect (Butzlaff, 2000). Therefore, evidence of a causal relationship between music training and verbal abilities is not yet clear and further exploration is merited.

Executive functioning is the generic term for multiple cognitive functions that use planning, organizing, and action to achieve future goals, including inhibition, updating/WM, and shifting (Miyake et al., 2000). Previous studies have shown that executive functions are related to music training (Bugos et al., 2007; Bialystok and DePape, 2009; Degé et al., 2011; Moreno et al., 2011; Seinfeld et al., 2013; Roden et al., 2014). A cross-sectional study showed that duration of music lessons for 9–12-year-old children was significantly related to their executive functions (Degé et al., 2011). However, in Schellenberg's (2011) cross-sectional study, when comparing the executive functions between musically trained 9–12-year-old children and untrained children, there were no significant differences on all measures of executive functions except for Digit Span (WISC-III). The available evidence linking music training with executive functions remains unclear (Schellenberg, 2011); therefore, further research is necessary.

Processing speed is the pace that one performs a given problem or task, and is closely related to children's academic performance (Ueno et al., 2010; Cassidy et al., 2016). In an intervention study among older adults, participants were randomly divided to either a piano group or an untrained control group (Bugos et al., 2007). After 6 months of individualized piano instruction, only the piano group significantly improved in processing speed (Digit Symbol). However, to date, there is few empirical evidence concerning the link between instrumental training and processing speed in children.

In the present study, we conducted an exploratory RCT to investigate the influence of short-term instrumental training program on children's cognitive functions including verbal ability, executive functions (WM and inhibition), and processing speed. To this end, we used four subtests (Vocabulary, Digit Span, Letter-Number Sequencing, and Digit Symbol) from the WISC-IV (Wechsler, 2003), as well as other cognitive tests, i.e., Rapid Automatic Naming (RAN) test (see Wagensveld et al., 2013) and the Go/No-go task (see Moreno et al., 2011).

## Methods

### Participants

Fifty-four children (aged 6–8-years-old) from a public elementary school in Kumamoto, Japan were recruited to take part in this study. Children's academic performance and personal details were assessed prior to the experiment. Academic achievement was acquired by their teacher's assessment. Personal details including age, sex, out of the school activities, and music background were acquired through parents' questionnaires. Fourteen children were excluded because they were currently participating in piano lessons. Therefore, forty children [22 boys and 18 girls; mean age = 7.49 years, standard deviation (SD) = 0.58] completed the experiment.

Participants were quasi-randomly assigned to an experimental group (*n* = 20; 11 boys and 9 girls) or a control group (*n* = 20; 11 boys and 9 girls); that is, after a completely random assignment, some adjustments were added to ensure that there were no differences between the two groups on the demographic variables as well as academic achievement before the instrumental intervention. The experimental group received a 6-week (12 sessions) music curriculum of playing the keyboard harmonica. The control group received no lesson (they only played during their lunch break where the experimental group received the keyboard harmonica lessons).

The Ethics Committee of Kumamoto University approved this study. Informed consent was obtained from all the parents in writing and verbal consent was obtained from the children.

### Musical intervention

Children in the experimental group were divided into two classes with ten children per class based on school grade. One teacher and two teaching assistants directed each class. The music curriculum was administered in separate classrooms at the same time (during the lunch break). Both classes received the same curriculum in two sessions in a week, resulting in 12 sessions for 6 weeks (25 min/session).

In Japan, because keyboard harmonica is widely used in music lessons from grader 3 on, almost every school child has a keyboard harmonica. Therefore, we chose the keyboard harmonica as the intervening instrument. In addition, previous results of our group using the keyboard harmonica showed that using music scores to teach novice may improve episodic memory, but obscure effects on executive function which has been often reported (Wada et al., 2017). Based on these results, we focused on improving children's executive functioning by modifying the intervention method. Therefore, we emphasized creating an association between sound and finger movement. These activities included singing and rhythm striking of small parts of tunes before playing the tunes with the keyboard harmonica, and playing the instrument while walking without seeing the keyboard. At first, children were guided to a series of music activities to make them experience various sounds emitted by the various keys on the instrument. They were asked to change their body posture in response to sounds of the instrument, such as jumping for the high sounds and crouching down for the low sounds, gradually crouching for a lowering series of sounds, and gradually reaching up for a rising series of sounds, and so on. Then, children were taught to play two familiar songs: “Antagata Dokosa (Where are you from?)” and “Jingle Bells” on the keyboard harmonica, without using a music score. These songs are composed of four or five musical notes that do not require transfer of the hand, which is good to play the instrument without looking at the keyboard, ultimately encouraging to play it while marching.

Specifically, learning to play a song on the keyboard harmonica comprised 3 stages. In the first stage, while listening to the song played by the teacher, the children attempted to sing with the lyrics. Then, for small sections of the song, the children were taught to sing with the melody (by using note names) while playing the rhythm with their hands. Finally, the children repeated and remembered the melody. In the second stage, the children were taught to play the melody on the keyboard harmonica, which was placed horizontally. In the third stage, the children were taught to play the melody on the keyboard harmonica, which was held vertically; therefore, visual cues for the hand and keys were unavailable. If children passed all the preceding procedures, they practiced playing the keyboard harmonica while marching.

### Measures

Before and after the instrumental intervention, both groups were administered a series of cognitive measurements. Most were subtests from the Japanese WISC-IV (Wechsler, 2003; Ueno et al., 2010), which is an intelligence test for children aged 5 years 0 months to 16 years 11 months. A high correlation between the Japanese version of the WISC-IV and existing tests including the WISC-III, WAIS-III, DN-CAS, and K-ABC has been confirmed by validity verification (Ueno et al., 2010). The Vocabulary, Digit Span, Letter-Number Sequencing, and Digit Symbol subtests of Japanese WISC-IV were administered, and the retest reliabilities were r = 0.80, r = 0.87, r = 0.67, and r = 0.84, respectively. Each subtest was concise, thus suitable to do in a brief time (i.e., students' lunch break). In addition, we added the RAN test (see Wagensveld et al., 2013) to assess verbal ability and the Go/No-go task based on a previous study (Moreno et al., 2011) to assess executive functions (inhibitory control).

#### Verbal ability measures

*Vocabulary* is a subtest of the Japanese version of the WISC-IV (Wechsler, 2003; Ueno et al., 2010), which was used to assess language development level, vocabulary knowledge, and formation of language concepts. The test consists of a picture task and a word task. In the picture task, children were asked to answer the name of the presented pictures. In the word task, children were asked to define a word (e.g., What is a hat?). The test was completed when children failed five consecutive questions.

*RAN* (Norton and Wolf, 2012) was used to measure processing speed of naming. This test requires children to name the array containing 5 repeated pictures (shoe, horse, house, dog, pot) and as many as possible within 1 min. The score was obtained from the number of pictures correctly named in 1 min.

#### Processing speed measures

*Digit symbol* is a subtest of the Japanese version of the WISC-IV (Wechsler, 2003; Ueno et al., 2010), which is used to measure processing speed and visual short-term memory. This test contains 9 pairs of numbers and symbols and asks children to copy as many of the symbols as possible corresponding to each number from 1–9 in 2 mins. The score was obtained from the number of correct answers in 2 mins.

#### WM measures

*Digit span* is a subtest of the Japanese version of the WISC-IV (Wechsler, 2003; Ueno et al., 2010), which consists of DSF and DSB, and is used to assess auditory WM, attention, and concentration. This test requires children to recall a sequence of digits in the same order (DSF) or in reverse order (DSB) that the experimenter aurally given them. For both DSF and DSB, digit sequences start from two numbers, and sequences increased when the children completed at least one of two trials of the same length correctly. The test was completed when children failed both trials of the same length. The score was obtained from the number of correctly recalled.

*Letter-Number Sequencing* is a subtest of the Japanese version of the WISC-IV and is used to measure auditory short-term memory, attention, and the ability to process two or more of information (numbers, letters) simultaneously (Wechsler, 2003; Ueno et al., 2010). Children were asked to reconstruct the sequences where combinations of numbers and letters (in kana) were aurally given by the experimenter via putting numbers in ascending order and letters in alphabetical order (e.g., “
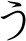
·4·3” → “3·4·
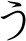
”). This test was completed when children failed three trials of the same length. The score was obtained from the number of correct answers.

#### Inhibitory control measures

The *Go/No-go task* paradigm was used to assess the ability to take appropriate actions (go) or to suppress the behavior (no-go) depending on the situation. In this study, the Go/No-go task was controlled by a computer and a program created by using PsychoPy (Version 1.85.2; Peirce, 2007). Stimuli were four geometric shapes (white triangle, purple triangle, white square, purple square), which were presented in random order on the screen. Children were asked to press the space key when a white stimulus appeared on the screen (go trials), and not to press the key when a purple stimulus appeared on the screen (no-go trials). Two types of geometric shapes were used to avoid repetition effects of the same color-shape pairing (Moreno et al., 2011). In each trail, first, a white plus sign appeared in the middle of the black screen for the duration varied randomly from 500 to 1,000 ms; then, the geometry stimulus appeared on the screen for a maximum of 1 s. Children were instructed to press the space key on go trials, and not to press the key on no-go trials. Next, a screen with a black background appeared for 500 ms as the inter-trial-interval. Response correctness (go trails and no-go trails) and the reaction time (go trails) were recorded by PsychoPy.

Children first completed 8 practice trials (go trials: 4, no-go trails: 4); then, they completed 40 test trials (go trials: 20, no-go trials: 20) that consisted of 20 trials × 2 blocks. Go trials and no-go trials were presented randomly on both practice trials and test trials.

### Procedure

Children were tested individually in a quiet classroom and all tests were conducted during a single session lasting approximately 30 min. During this session, the children completed the Go/No-go task, RAN test, and four subtests (Digit span, Digit symbol, Vocabulary, Letter-Number Sequencing) of the Japanese version of the WISC-IV (Ueno et al., 2010). Assistants were trained in administering these cognitive measurements and were blind to type of group (experimental or control) during the data collection. Before and after the instrumental intervention, these tests were administered in the same order for each child. In addition, to avoid a time conflict between the instrumental intervention and school events, the pre-test (September 13–21, 2016) was conducted slightly ahead of the 6-week instrumental intervention (November 1–December 7, 2016). After the instrumental intervention, the post-test was conducted December 8–16, 2016.

### Data analyses

Demographic data and pre-test scores were analyzed using independent *t*-tests between the two groups (experimental group and control group) to evaluate whether the quasi-random grouping resulted in homogeneous groups. To test an intervention effect of instrumental music training, first, we calculated the distribution of all datasets via the Kolmogorov-Smirnov test. The distribution for normality was not confirmed for some datasets; therefore, we used a two-way mixed analyses of variance (ANOVA) with the permutation non-parametric method (as used in Neuroimaging analysis; Nichols and Holmes, 2002). We used a random re-sampling method to avoid a multiple testing problem or type I error. The permutation test obtains a testing distribution of statistical scores such as F-values just by multiplicatively performing tests with re-sampled data, and calculates the statistical threshold, which accidentally yields the significant α level (e.g., *p* < 0.05) under a multiple testing condition.

First, we transformed raw data (2 groups × 2 times × 20 participants) into z-scores within each of the 10 cognitive measurements, because they possessed non-uniform and diverse types of scale ranges and intervals. Transformed data were combined into single datasets (2 groups × 2 times × 20 participants × 10 tasks) and were randomly re-sampled to build a dummy two-way mixed ANOVA data with the between-participants factor of group (control, intervention) and the within-participants factor of time (pre- and post-intervention). Such random re-sampling and successive two-way ANOVAs were recursively conducted 1,000 times to obtain dummy F-values for the main effects of group and time, as well as the interaction effect. Finally, actual F-values for each cognitive measurement were calculated and tested against permutation distributions of dummy 1,000 F-values based on the criterion of the corrected α level of pc < 0.05. This permutation test was conducted by a program written by MATLAB (Mathworks, Natick, USA).

## Results

### Musical intervention

All children (*n* = 20) in the experimental group could play “*Antagata Dokosa*” (phrases 1–3) and “*Jingle Bells*” (phrases 1–4) (see Supplementary Material). Twelve children could play the keyboard harmonica while walking and holding the instrument vertically; two children could play the keyboard harmonica, which was held vertically; five children could play the instrument, which was placed horizontally; and one child with learning difficulties could play the keyboard harmonica placed horizontally while looking at note names on the memo.

### Demographics and pre-test

All children completed the study. The average attendance rate was 90%. There were no significant differences between the two groups in both children's age and academic performance. At the pre-test, there were no significant differences between the two groups on the cognitive measurements (Table 2), indicating the two groups were equivalent.

**Table 2:** Mean (standard deviation) of demographic and pre-test performance for the experimental group and control group.

	**Experimental (*n* = 20)**	**Control (*n* = 20)**
Number of boys/girls	11/9	11/9
Grade 1/2	10/10	9/11
	Mean (standard deviation)	Mean (standard deviation)
Age (years)	7.50 (0.59)	7.48 (0.58)
Academic performance	2.2 (0.51)	2.175 (0.69)
Vocabulary	16.6 (4.25)	15.85 (4.95)
Rapid automatic naming	49.15 (10.74)	51.2 (8.23)
Digit symbol	34.75 (9.22)	33.85 (7.18)
Digit span	12.6 (2.13)	12.65 (1.98)
Letter-number sequencing	12.05 (3.63)	12.6 (3.81)
Go task (reaction time)	0.55 (0.08)	0.53 (0.10)
Go task (correct)	0.96 (0.05)	0.97 (0.06)
No-go task (correct)	0.94 (0.06)	0.91 (0.09)

### Intervention effects

#### WM

**Figure 1:**
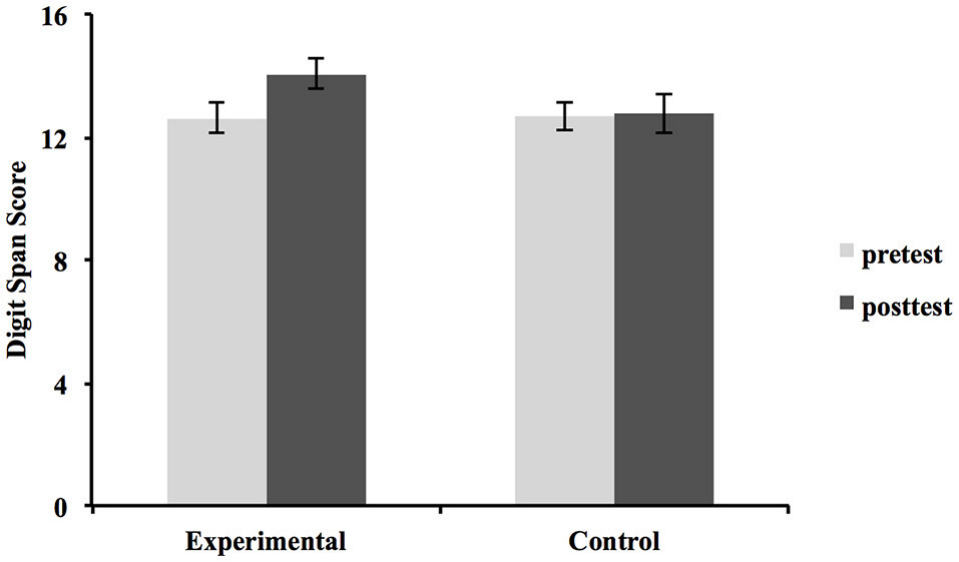
Results of Digit Span total raw scores. Error bars indicate standard errors of the mean.

**Figure 2:**
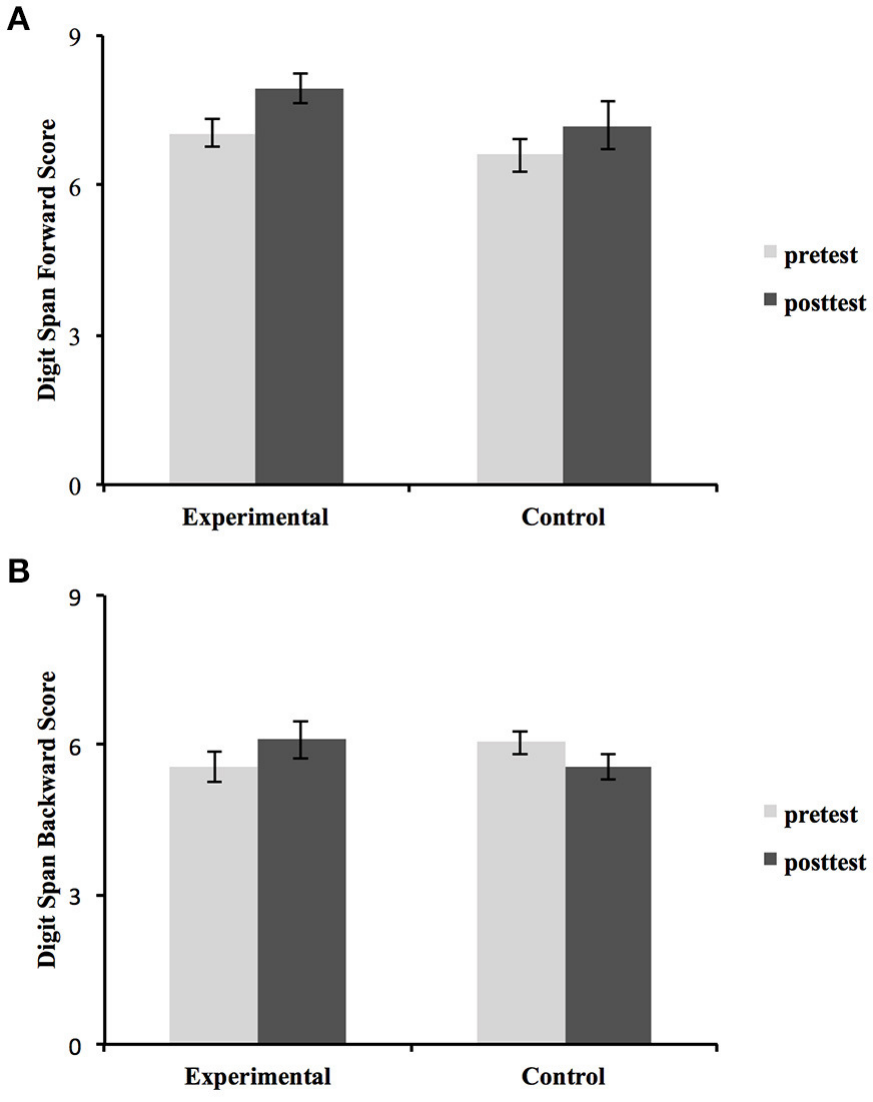
**(A)** Results of Digit Span Forward raw scores; **(B)** Results of Digit Span Backward raw scores. Error bars indicate standard errors of the mean.

The Group × Time ANOVA of the total Digit Span score showed a significant main effect of Time [*F*_(1, 38)_ = 5.977, *p* = 0.017, $\eta_{\text{p}}^{2}$ = 0.136] and a Group × Time interaction [*F*_(1, 38)_ = 4.534, *p* = 0.034, $\eta_{\text{p}}^{2}$ = 0.107]. This interaction was supposed to represent the effect of intervention (Figure 1). Interestingly, further ANOVAs on Digit Span (Forward and Backward separately) showed no significant interaction on DSF [*F*_(1, 38)_ = 0.458, *p* = 0.491, $\eta_{\text{p}}^{2}$ = 0.012], but a significant Group × Time interaction on DSB [*F*_(1, #38)_ = 6.353, *p* = 0.015, $\eta_{\text{p}}^{2}$ = 0.143]. On the other hand, a significant main effect of Time was found for the DSF [*F*_(1, 38)_ = 11.461, *p* = 0.002, $\eta_{\text{p}}^{2}$ = 0.232]. No significant Time effect was found for DSB. Figure 2A indicates that the scores of DSF increased for both groups. As shown in Figure 2B, the DSB scores of the experimental group increased after intervention, while the control group's scores did not increase over time.

In the Letter-Number Sequencing, we found a significant main effect of Time [*F*_(1, 38)_ = 5.451, *p* = 0.024, $\eta_{\text{p}}^{2}$ = 0.125]. There was no significant Group × Time interaction [*F*_(1, 38)_ = 0.003, *p* = 0.869, $\eta_{\text{p}}^{2}$ < 0.001]. Therefore, while both groups had increased scores from the pre-test, no intervention effect was found.

#### Inhibitory control

In the Go/No-go task, the results indicated a ceiling effect. Both the experimental (Go task: pre-test mean ± SD = 96% ± 0.05%; post-test mean ± SD = 97% ± 0.04%; No-go task: pre-test mean ± SD = 94% ± 0.06; post-test mean ± SD = 95% ± 0.08) and control groups (Go task: pre-test mean ± SD = 97% ± 0.06; post-test mean ± SD = 98% ± 0.05; No-go task: pre-test mean ± SD = 91% ± 0.09; post-test mean ± SD = 93% ± 0.10) showed a correct rate over 90% before and after instrumental intervention. No significant main effects or interaction were found on correct rate of the Go/No-go task. Moreover, analysis of reaction times showed no significant main or interaction effects.

#### Verbal ability and processing speed

The group by time interaction, as a measure of the intervention effect, was not significant in any of tests measuring verbal ability and processing speed (Vocabulary: [*F*_(1, #38)_ = 1.327, *p* = 0.248, $\eta_{\text{p}}^{2}$ = 0.034]; RAN: [*F*_(1, 38)_ = 1.562, *p* = 0.212, $\eta_{\text{p}}^{2}$ = 0.039], and Digit Symbol: [*F*_(1, 38)_ = 0.188, *p* = 0.666, $\eta_{\text{p}}^{2}$ = 0.005]). A significant main effect of Time was found on the RAN [*F*_(1, 38)_ = 42.69, *p* < 0.001, $\eta_{\text{p}}^{2}$ = 0.529] and Digit Symbol [*F*_(1, 38)_ = 68.91, *p* < 0.001, $\eta_{\text{p}}^{2}$ = 0.645], indicating both groups' improvement over time. No significant main effect was found for Vocabulary test. The means (SD) of all cognitive measurements data, *F*-values and *p*-values from the ANOVA with a permutation test are shown in Table 3 (the *F*-value distribution of the ANOVA with the permutation test are shown in Supplementary Material).

**Table 3:** The means (SD) of all cognitive measurements data, and *F*-values and *p*-values from the ANOVA with a permutation test.

**Measures**	**Experimental (*****n*** = **20)**		**Control (***n*** = 20)**		**Group** ^*^ **Time**		**Group**		**Time**	
	**Pre-test**	**Post-test**	**Pre-test**	**Post-test**	***F*-value (1,38)**	***p*-value**	***F*-value (1,38)**	***p*-value**	***F*-value (1,38)**	***p*-value**
Vocabulary (RS)	16.6 (4.25)	17.1 (3.82)	15.85 (4.95)	17.9 (3.00)	1.327	0.248	0	≈1	3.592	0.07
RAN (RS)	49.15 (10.74)	56 (12.13)	51.2 (8.23)	55.85 (9.34)	1.562	0.212	0.088	0.756	42.69	< 0.001
Digit symbol (RS)	34.75 (9.22)	41.3 (8.87)	33.85 (7.18)	39.75 (8.20)	0.188	0.666	0.218	0.63	68.91	< 0.001
Digit span (RS)	12.6 (2.13)	14.05 (2.09)	12.65 (1.98)	12.75 (2.83)	4.534	0.034	0.874	0.341	5.977	0.017
DSF (RS)	7.05 (1.20)	7.95 (1.32)	6.6 (1.43)	7.2 (2.14)	0.458	0.491	1.725	0.172	11.461	0.002
DSB (RS)	5.55 (1.32)	6.1 (1.61)	6.05 (1.02)	5.55 (1.12)	6.353	0.015	0.005	0.941	0.014	0.901
LNS (RS)	12.05 (3.63)	13.05 (3.02)	12.6 (3.81)	13.65 (4.20)	0.003	0.869	0.267	0.609	5.451	0.024
Go (correct)	0.96 (0.05)	0.97 (0.04)	0.97 (0.06)	0.98 (0.05)	0.011	0.923	0.106	0.744	0.556	0.462
Go (RT)	0.55 (0.08)	0.53 (0.08)	0.53 (0.10)	0.51 (0.04)	0.035	0.858	0.757	0.381	1.618	0.205
No-go (correct)	0.94 (0.06)	0.95 (0.08)	0.91 (0.09)	0.93 (0.10)	0.084	0.773	0.927	0.332	0.458	0.496

*RS, Raw Score; RAN, Rapid Automatic Naming; DSF, Digit Span Forward; DSB, Digit Span Backward; LNS, Letter-Number Sequencing*.

## Discussion

This exploratory pilot study investigated the effect of 6-weeks of musical instrumental training on verbal ability, WM, inhibition, and processing speed in children. Our results showed a significant group by time interaction in the Digit Span Test, indicating that Digit Span total scores significantly increased in the experimental group, whereas such a change was not found in the control group. In addition, after the instrumental training, the mean scaled score, which is a standardized score (with a mean of 10 and a standard deviation of 3) obtained based on the test results of children within the same age group (Ueno et al., 2010) increased from 9.4 to 10.95 in the experimental group and from 9.55 to 9.7 in the control group. In only 6 weeks, the experimental group improved their relative position in the normal distribution by 20% (1.55/3 SD). These preliminary results indicate that playing a musical instrument may be beneficial toward improving children's WM. The retest reliability of Digit Span (*r* = 0.87; Ueno et al., 2010) used may also strengthen the reliability of our results to some extent, however, no significant influences were found on the other cognitive tests.

A cross-sectional study showed that musically trained 9- to 12-year-old children outperformed untrained children on the Digit Span Test (Schellenberg, 2011). Similar with this finding, the present study showed that 6-weeks of instrumental training significantly improved children's Digit Span scores. Since our instrumental training curriculum did not use the musical score, this improvement suggests a strengthened sensorimotor association (auditory and somato-motor). Moreover, to be able to play the song on the keyboard without musical score, children must remember the heard melodies and rhythms. For that reason, children must concentrate on listening to melodies and rhythms, then repeat them many times until they learn them by heart. Furthermore, to be able to play the keyboard harmonica while marching, children must actively allocate attentional resources to the instrumental performance and march. These instructional methods might have been effective at improving auditory WM.

Since DSF and DSB subtests of Digit Span emphasize distinct aspects of WM (phonological loop and central executive, respectively) (Davis and Pratt, 1995), we analyzed the two subtests separately. Results from the DSF subtest showed no significant interaction, indicating no significant effect of short-term instrumental training on the phonological loop of WM (DSF). In contrast, Roden et al. (2014) found that children who received 18 months of instrumental music lessons had superior performance on the phonological loop of WM than those received natural science lessons; Moreover, Lee et al. (2007) showed that the children who had an average of 6.1 years of music experience had higher DSF scores than children without music experience. Note that the duration of our instrumental training curriculum (6 weeks) was substantially shorter than both Roden et al. (2014) (18 months) and Lee et al. (2007). Therefore, we speculate that these difference results might be due to differences in the training duration.

On the other hand, concerning DSB, as a measure of central executive (Davis and Pratt, 1995), the significant Group × Time interaction indicated a positive effect of our instrumental training curriculum. This result was similar with a few previous studies (Lee et al., 2007; Roden et al., 2014). A cross-sectional study showed that musically trained children performed better than untrained children on the central executive of WM (Lee et al., 2007). Moreover, even a longitudinal study measuring WM by other measurements showed that the central executive of WM was improved in children after 18 months of instrumental training (Roden et al., 2014).

In contrast, no significant intervention effect was found in the Letter-Number Sequencing test that was also used to measure WM. We speculate that it might be because Letter-Number Sequencing has a higher manipulation load than Digit Span, since Letter-Number Sequencing test requires not only information storage and retrieval, but also processing of two or more of information (numbers, letters) at the same time (Ueno et al., 2010). Another possible explanation for this result is that since the participants were the 1st and 2nd graders in a primary school, they may not yet be proficient in the Japanese alphabetical order.

Concerning verbal ability, our results showed no effect of instrumental training on the Vocabulary test. However, some previous studies (Schellenberg, 2004, 2006; Forgeard et al., 2008; Moreno et al., 2011) showed associations between music training and improvements of vocabulary comprehension. For example, Schellenberg (2004) showed that the children who received 9 months of keyboard or vocal lessons showed a significant improvement in general intelligence that contains vocabulary tests. Forgeard et al. (2008) showed that the children who received music lessons for 3 years or more had higher vocabulary scores than children who had not received music lessons, and the duration of music lessons could predict the result of vocabulary subtest. Therefore, we speculate that the difference in the above findings might be due to differences in the duration of music training, and short-term instrumental training might not improve vocabulary comprehension.

Concerning inhibition, we failed to replicate the previous Go/No-go results of Moreno et al. (2011). However, the children (6–8-years-old) in our study were older than the children (4–6-years-old) in that study. Perhaps this task was too simple for 6–8-year-old children that it caused a ceiling effect (both groups showed a correct rate over 90% before and after the musical intervention). Therefore, adjusting the degree of difficulty of the test is also vital. Moreover, the musical intervention used in a previous study (Moreno et al., 2011) was a music-listening training based on a computer and was not instrumental training. Differences in the training method might produce inconsistent results.

Finally, we should note some limitations of this study. One is the relatively small sample size (*N* = 40). Future RCT research should investigate the effects of short-term instrumental music training on WM with a larger sample size to obtain more generalizable results. Another limitation was that we used subtests of the WISC-VI (Wechsler, 2003) to assess a single cognitive skill. Relative to standalone cognitive measures, subtests might obtain less information on that cognitive skill (Mehr et al., 2013). In addition, we were unable to clarify whether the improvement of WM was due to the temporary effect caused by novelty, or if it will further improve with continuous practice. Finally, this study lacks a control group that received some attention from teachers, which limits the interpretation of findings. Future research should address these issues.

In summary, this exploratory study showed that short-term instrumental training (playing the keyboard harmonica) improved children's Digit Span scores (especially Digit Span Backward scores), and in only 6 weeks, the experimental group improved their relative position in the normal distribution by 20%. These preliminary results indicate that playing a musical instrument may be beneficial toward improving children's WM. Although the small effect size should be cautiously taken, this exploratory study provides the initial empirical data that plays a role for further research on the effects of relatively short-term instrumental music training on WM.

## Author contributions

XG, CO, AS, and KS conceived and designed the study. XG, CO, AS, and KS performed the experiments. XG and KS analyzed the data and wrote the paper. XG, CO, AS, and KS reviewed and edited the manuscript. All authors read and approved the manuscript.

### Conflict of interest statement

The authors declare that the research was conducted in the absence of any commercial or financial relationships that could be construed as a potential conflict of interest.

## References

[B1] Beggs D. L. Mouw J. T. (1980). Developing Cognitive Abilities Test. Carbondale, IL: Southern Illinois University Press.

[B2] Bengtsson S. L. Nagy Z. Skare S. Forsman L. Forssberg H. Ullén F. (2005). Extensive piano practicing has regionally specific effects on white matter development. Nat. Neurosci. 8, 1148–1150. 10.1038/nn1516 16116456

[B3] Bialystok E. Depape A. M. (2009). Musical expertise, bilingualism, and executive functioning. J. Exp. Psychol. 35, 565–574. 10.1037/a0012735 19331508

[B4] Bruininks R. H. (1978). Bruininks-Oseretsky Test of Motor Proficiency. Circle Pines, MN: American Guidance Center.

[B5] Bugos J. A. Perlstein W. M. McCrae C. S. Brophy T. S. Bedenbaugh P. H. (2007). Individualized piano instruction enhances executive functioning and working memory in older adults. Aging Ment. Health 11, 464–471. 10.1080/13607860601086504 17612811

[B6] Butzlaff R. (2000). Can music be used to teach reading? J. Aesthet. Educ. 34, 167–178. 10.2307/3333642

[B7] Canadian Test Centre (1992). Canadian Achievement Tests, 2nd edn. Markham, ON: Canadian Test Centre.

[B8] Cassidy A. R. White M. T. DeMaso D. R. Newburger J. W. Bellinger D. C. (2016). Processing speed, executive function, and academic achievement in children with dextro-transposition of the great arteries: testing a longitudinal developmental cascade model. Neuropsychology 30, 874–885. 10.1037/neu0000289 27077787 PMC5042819

[B9] Chan A. S. Ho Y. C. Cheung M. C. (1998). Music training improves verbal memory. Nature 396, 128–128. 10.1038/24075 9823892

[B10] Cheek J. M. Smith L. R. (1999). Music training and mathematics achievement. Adolescence 34, 759–761. 10730700

[B11] Chobert J. Marie C. François C. Schön D. Besson M. (2011). Enhanced passive and active processing of syllables in musician children. J. Cogn. Neurosci. 23, 3874–3887. 10.1162/jocn_a_00088 21736456

[B12] Coopersmith S. (1981). Coopersmith Self-Esteem Inventories. Palo Alto, CA: Consulting Psychologists Press.

[B13] Costa-Giomi E. (1999). The effects of three years of piano instruction on children's cognitive development. J. Res. Music Educ. 47, 198–212. 10.2307/3345779

[B14] Davis H. L. Pratt C. (1995). The development of children's theory of mind: the working memory explanation. Aust. J. Psychol. 47, 25–31. 10.1080/00049539508258765

[B15] Debowska W. Wolak T. Nowicka A. Kozak A. Szwed M. Kossut M. (2016). Functional and structural neuroplasticity induced by short-term tactile training based on Braille reading. Front. Neurosci. 10:460. 10.3389/fnins.2016.00460 27790087 PMC5061995

[B16] Degé F. Kubicek C. Schwarzer G. (2011). Music lessons and intelligence: a relation mediated by executive functions. Music Percept. 29, 195–201. 10.1525/mp.2011.29.2.195

[B17] Degé F. Schwarzer G. (2011). The effect of a music program on phonological awareness in preschoolers. Front. Psychol. 2:124. 10.3389/fpsyg.2011.00124 21734895 PMC3121007

[B18] Forgeard M. Winner E. Norton A. Schlaug G. (2008). Practicing a musical instrument in childhood is associated with enhanced verbal ability and nonverbal reasoning. PLoS ONE 3:e3566. 10.1371/journal.pone.0003566 18958177 PMC2570220

[B19] Fujioka T. Ross B. Kakigi R. Pantev C. Trainor L. J. (2006). One year of musical training affects development of auditory cortical-evoked fields in young children. Brain 129, 2593–2608. 10.1093/brain/awl247 16959812

[B20] Gaser C. Schlaug G. (2003). Brain structures differ between musicians and non-musicians. J. Neurosci. 23, 9240–9245. 14534258 10.1523/JNEUROSCI.23-27-09240.2003 PMC6740845

[B21] Gordon E. E. (1988). Musical Aptitude Profile. Chicago, IL: Riverside Publishing Co.

[B22] Gouzouasis P. Guhn M. Kishor N. (2007). The predictive relationship between achievement and participation in music and achievement in core grade 12 academic subjects. Music Educ. Res. 9, 81–92. 10.1080/14613800601127569

[B23] Hasselhorn M. Schumann-Hengsteler R. Gronauer J. Grube D. Mähler C. Schmid I. (2012). Arbeitsgedächtnistestbatterie für Kinder von 5–12 Jahren (AGTB 5–12) [Working Memory Test Battery for Children From 5–12 Years of Age (AGTB 5–12)]. Göttingen: Hogrefe.

[B24] Helmbold N. Rammsayer T. Altenmüller E. (2005). Differences in primary mental abilities between musicians and nonmusicians. J. Individ. Diff. 26, 74–85. 10.1027/1614-0001.26.2.74

[B25] Helmstaedter C. Lendt M. Lux S. (2001). Verbaler Lern und Merkfȧhigkeitstest (VLMT) [Audi- tory Verbal Learning Test]. Göttingen: Hogrefe.

[B26] Kaufman A. S. Kaufman N. L. (1985). Kaufman Test of Educational Achievement. Circle Pines, MN: American Guidance Service.

[B27] Keenan J. P. Thangaraj V. Halpern A. R. Schlaug G. (2001). Absolute pitch and planum temporale. Neuroimage 14, 1402–1408. 10.1006/nimg.2001.0925 11707095

[B28] Landi S. M. Baguear F. Della-Maggiore V. (2011). One week of motor adaptation induces structural changes in primary motor cortex that predict long-term memory one year later. J. Neurosci. 31, 11808–11813. 10.1523/JNEUROSCI.2253-11.2011 21849541 PMC3180815

[B29] Lee Y. S. Lu M. J. Ko H. P. (2007). Effects of skill training on working memory capacity. Learn. Inst. 17, 336–344. 10.1016/j.learninstruc.2007.02.010

[B30] Levitin D. J. (2006). This is Your Brain on Music: The science of a human obsession. New York, NY: Penguin.

[B31] Mehr S. A. Schachner A. Katz R. C. Spelke E. S. (2013). Two randomized trials provide no consistent evidence for nonmusical cognitive benefits of brief preschool music enrichment. PLoS ONE 8:e82007. 10.1371/journal.pone.0082007 24349171 PMC3859544

[B32] Miyake A. Friedman N. P. Emerson M. J. Witzki A. H. Howerter A. Wager T. D. (2000). The unity and diversity of executive functions and their contributions to complex “frontal lobe” tasks: a latent variable analysis. Cogn. Psychol. 41, 49–100. 10.1006/cogp.1999.0734 10945922

[B33] Moreno S. Bialystok E. Barac R. Schellenberg E. G. Cepeda N. J. Chau T. (2011). Short-term music training enhances verbal intelligence and executive function. Psychol. Sci. 22, 1425–1433. 10.1177/0956797611416999 21969312 PMC3449320

[B34] Nichols T. E. Holmes A. P. (2002). Nonparametric permutation tests for functional neuroimaging: a primer with examples. Hum. Brain Mapp. 15, 1–25. 10.1002/hbm.1058 11747097 PMC6871862

[B35] Norton E. S. Wolf M. (2012). Rapid automatized naming (RAN) and reading fluency: implications for understanding and treatment of reading disabilities. Annu. Rev. Psychol. 63, 427–452. 10.1146/annurev-psych-120710-100431 21838545

[B36] Peirce J. W. (2007). PsychoPy-psychophysics software in Python. J. Neurosci. Methods 162, 8–13. 10.1016/j.jneumeth.2006.11.017 17254636 PMC2018741

[B37] Piro J. M. Ortiz C. (2009). The effect of piano lessons on the vocabulary and verbal sequencing skills of primary grade students. Psychol. Music 37, 325–347. 10.1177/0305735608097248

[B38] Reynolds C. R. Kamphaus R. W. (1992). Behavior Assessment System for Children. Circle Pines, MN: American Guidance Service.

[B39] Roden I. Grube D. Bongard S. Kreutz G. (2014). Does music training enhance working memory performance? Findings from a quasi-experimental longitudinal study. Psychol. Music 42, 284–298. 10.1177/0305735612471239

[B40] Roden I. Kreutz G. Bongard S. (2012). Effects of a school-based instrumental music program on verbal and visual memory in primary school children: a longitudinal study. Front. Psychol. 3:00572. 10.3389/fpsyg.2012.00572 23267341 PMC3528082

[B41] Schellenberg E. G. (2004). Music lessons enhance IQ. Psychol. Sci. 15, 511–514. 10.1111/j.0956-7976.2004.00711.x 15270994

[B42] Schellenberg E. G. (2006). Long-term positive associations between music lessons and IQ. J. Educ. Psychol. 98, 457–468. 10.1037/0022-0663.98.2.457

[B43] Schellenberg E. G. (2011). Examining the association between music lessons and intelligence. Br. J. Psychol. 102, 283–302. 10.1111/j.2044-8295.2010.02000.x 21751987

[B44] Schlaug G. Jäncke L. Huang Y. Staiger J. F. Steinmetz H. (1995a). Increased corpus callosum size in musicians. Neuropsychologia 33, 1047–1055. 8524453 10.1016/0028-3932(95)00045-5

[B45] Schlaug G. Jäncke L. Huang Y. Steinmetz H. (1995b). *In vivo* evidence of structural brain asymmetry in musicians. Science 267, 699–701. 7839149 10.1126/science.7839149

[B46] Schneider P. Sluming V. Roberts N. Scherg M. Goebel R. Specht H. J.. (2005). Structural and functional asymmetry of lateral Heschl's gyrus reflects pitch perception preference. Nat. Neurosci. 8, 1241–1247. 10.1038/nn1530 16116442

[B47] Scholz J. Klein M. C. Behrens T. E. Johansen-Berg H. (2009). Training induces changes in white-matter architecture. Nat. Neurosci. 12, 1370–1371. 10.1038/nn.2412 19820707 PMC2770457

[B48] Seinfeld S. Figueroa H. Ortiz-Gil J. Sanchez-Vives M. V. (2013). Effects of music learning and piano practice on cognitive function, mood and quality of life in older adults. Front. Psychol. 4:810. 10.3389/fpsyg.2013.00810 24198804 PMC3814522

[B49] Sekiyama K. Miyauchi S. Imaruoka T. Egusa H. Tashiro T. (2000). Body image as a visuomotor transformation device revealed in adaptation to reversed vision. Nature 407, 374–377. 10.1038/35030096 11014192

[B50] Sluming V. Barrick T. Howard M. Cezayirli E. Mayes A. Roberts N. (2002). Voxel-based morphometry reveals increased gray matter density in Broca's area in male symphony orchestra musicians. Neuroimage 17, 1613–1622. 10.1006/nimg.2002.1288 12414299

[B51] Stoesz B. M. Jakobson L. S. Kilgour A. R. Lewycky S. T. (2007). Local processing advantage in musicians: Evidence from disembedding and constructional tasks. Music Percept. 25, 153–165. 10.1525/mp.2007.25.2.153

[B52] Ueno K. Fujita K. Maekawa H. Ishikuma T. Dairoku H. Matsuda O. (2010). Japanese Version of Wechsler Intelligence Scale for Children, 4th Edn. Tokyo: Nihon Bunka Kagakusha.

[B53] Wada R. Hisanaga S. Kaku K. Kimura H. Suzuki M. Kawagoe T. (2017). The effects of playing the keyboard harmonica on older adults verbal memory, in Paper presented at the 15th World Congress of Music Therapy, Tsukuba.

[B54] Wagensveld B. van Alphen P. Segers E. Hagoort P. Verhoeven L. (2013). The neural correlates of rhyme awareness in preliterate and literate children. Clin. Neurophysiol. 124, 1336–1345. 10.1016/j.clinph.2013.01.022 23523114

[B55] Wechsler D. (1991). Wechsler Intelligence Scale for Children–3rd Edn. San Antonio, TX: Psychological Corporation.

[B56] Wechsler D. (1999). Wechsler Abbreviated Scale of Intelligence. San Antonio, TX: Psychological Corporation.

[B57] Wechsler D. (2003). Wechsler intelligence scale for children-−4th Edn. London: Pearson.

[B58] Zatorre R. J. Perry D. W. Beckett C. A. Westbury C. F. Evans A. C. (1998). Functional anatomy of musical processing in listeners with absolute pitch and relative pitch. Proc. Natl. Acad. Sci. U.S.A. 95, 3172–3177. 10.1073/pnas.95.6.3172 9501235 PMC19714

