# Supplementary material for: Improved Digit Span in Children after a 6-Week Intervention of Playing a Musical Instrument: An Exploratory Randomized Controlled Trial
Source: Front Psychol. 2018 Jan 8;8:2303. doi: 10.3389/fpsyg.2017.02303 (PMC5766672; doi:10.3389/fpsyg.2017.02303)

Supplementary Material

Improved Digit Span in Children after a 6-Week Intervention of Playing a Musical Instrument: An Exploratory Randomized Controlled Trial

Xia Guo, Chie Ohsawa, Akiko Suzuki and Kaoru Sekiyama*

*** Correspondence:** Kaoru Sekiyama: sekiyama.kaoru.8a@kyoto-u.ac.jp

1 Target songs: '*Antagata Dokosa*' and '*Jingle Bells*'

Antagata Dokosa

la la la do | re do re | mi do re | Phrase 1

mi mi re do la | la do do do re | Phrase 2

do re mi mi | re do la | re do la ‖ Phrase 3

Jingle Bells

mi mi mi – | mi mi mi – | mi sol do re | mi – – – | Phrase 1

fa fa fa fa | fa mi mi mi | mi re re do | re – sol – | Phrase 2

mi mi mi – | mi mi mi – | mi sol do re | mi – – – | Phrase 3

fa fa fa fa | fa mi mi mi | sol sol fa re | do – – – ‖ Phrase 4

Figure S1. F-value distribution of an analysis of variance with the permutation test

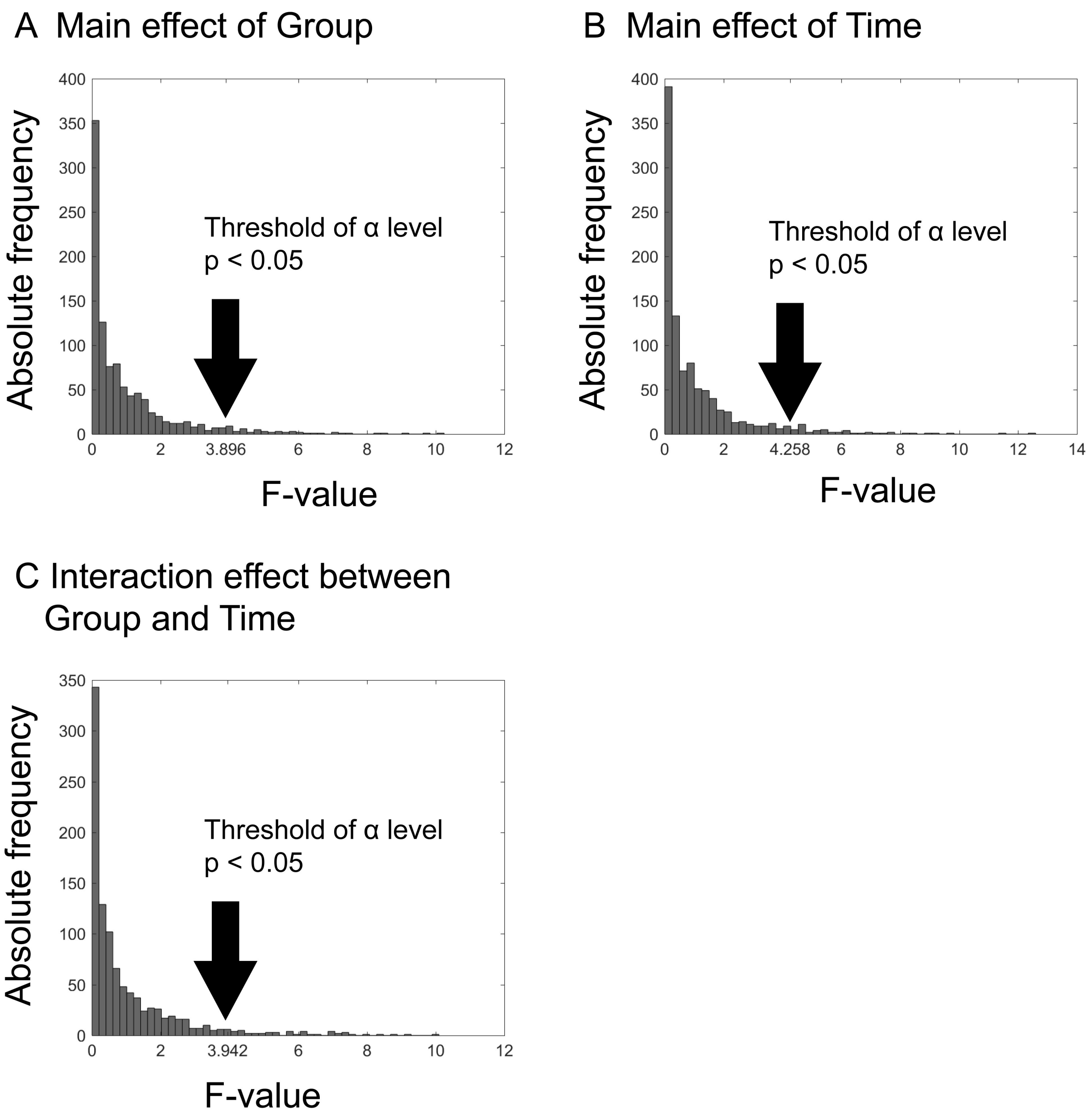

Supplement: Supplementary file 1 [file Table1.pdf]
